# Fluoxetine disrupts cholesterol metabolism in endothelial cells via SREBP2 activation

**DOI:** 10.1038/s41398-026-04197-x

**Published:** 2026-06-23

**Authors:** Fabiana Oliveira, Christina Papa, Tobias Hagemann, Ruby Schipper, Florian Geier, Tino Röxe, Faiqa Zulfqar, Christoph Prönnecke, Lisa Schmidt, Hryhoriy Stryhanyuk, Anne Hoffmann, Anastasia Kyselova, Christina Karantanou, Yuli Buckley, Muhammad Asad Farhan, Jesús Rafael Rodríguez-Aguilera, Saira Ambreen, He Yao, Amna Arif, Hugo N. G. Martin, Thomas Ebert, Nora Klöting, Matthias Blüher, Khurrum Shahzad, Jes-Niels Boeckel, Carolina E. Hagberg, Rima Chakaroun, Sofia-Iris Bibli, Bilal N. Sheikh

**Affiliations:** 1 Helmholtz Institute for Metabolic, Obesity and Vascular Research (HI-MAG) of the Helmholtz Center Munich, Leipzig, Germany; 2 Faculty of Medicine, University of Leipzig, Leipzig, Germany; 3 Department of Medicine Solna, Division of Cardiovascular Medicine, Karolinska Institutet, Stockholm, Sweden; 4 Center for Molecular Medicine, Karolinska University Hospital, Stockholm, Sweden; 5 Klinik und Poliklinik für Kardiologie, Universitätsklinikum Leipzig, Leipzig, Germany; 6 Department of Technical Biogeochemistry, Helmholtz Centre for Environmental Research - UFZ, Leipzig, Germany; 7 European Center for Angioscience, Department of Vascular Dysfunction, Medical Faculty Mannheim, Heidelberg University, Mannheim, Germany; 8 Institute of Laboratory Medicine, Clinical Chemistry, and Molecular Diagnostics, University Hospital Leipzig, Leipzig, Germany; 9 Medical Department III - Endocrinology, Nephrology, Rheumatology, University of Leipzig Medical Center, Leipzig, Germany; 10 Wallenberg Laboratory, Department of Molecular and Clinical Medicine, Institute of Medicine, University of Gothenburg, Gothenburg, Sweden; 11 German Centre for Cardiovascular Research (DZHK), Berlin, Germany; 12 Helmholtz-Institute for Translational AngioCardioScience (HI-TAC) of the Max Delbrück Center for Molecular Medicine in the Helmholtz Association (MDC), Heidelberg University, Heidelberg, Germany; 13 German Centre for Diabetes Research (DZD), Neuherberg, Germany

**Keywords:** Physiology, Diseases, Pathogenesis

## Abstract

Fluoxetine is a selective serotonin reuptake inhibitor (SSRI) commonly prescribed for the treatment of depressive disorders. Recent clinical reports and studies in animal models have suggested that fluoxetine increases the risk of cardiovascular diseases, but the underlying mechanisms remain unknown. Here, we uncover that fluoxetine disrupts lipid and cholesterol metabolism in primary human endothelial cells (ECs). Fluoxetine triggered an upregulation of cholesterol metabolism genes, leading to the accumulation of lipid droplets in ECs. We find higher levels of cholesterol esters, ceramides, sphingolipids and fatty acids in ECs treated with fluoxetine. The disruption of lipid homeostasis was driven by increased cholesterol biosynthesis, as well as low-density lipoprotein (LDL) uptake and transcytosis via the LDL receptor. Fluoxetine accumulated in ECs in the endoplasmic reticulum (ER), caused ER expansion and reduced protein translation, without inducing ER stress markers. Mechanistically, fluoxetine activated the SREBP2 transcription factor in an INSIG-dependent manner. SREBP2 inhibition attenuated the fluoxetine-mediated upregulation of the LDL receptor and lipid accumulation. Our findings reveal that fluoxetine reprograms lipid metabolism and leads to endothelial dysfunction.

## Introduction

Depressive disorders are the second most common disabling condition amongst adults with roughly 280 million cases worldwide [1, 2]. The two main treatments used in their management are psychotherapy and pharmacotherapy [3]. Selective serotonin reuptake inhibitors (SSRIs), including sertraline, fluoxetine and citalopram, are presently recommended as the first-line of treatment for depression [4]. In multiple countries, fluoxetine (brand name Prozac) was the first SSRI approved for clinical use. More than 40 million patients have been prescribed fluoxetine since the 1980s [5, 6], and it is still widely used.

Recent clinical studies have associated fluoxetine with adverse cardiovascular events. This includes an association with an increased risk of myocardial infarction and stroke [7, 8]. In addition to clinical analyses, the impact of fluoxetine on cardiovascular disease (CVD) has also been analysed in animal models. In the atherosclerosis-prone *Apoe* knockout mice, fluoxetine increases early plaque formation independently of its well-established role in inhibiting serotonin reuptake [9]. However, the underlying mechanisms remain unknown. Understanding how fluoxetine mechanistically impacts vascular health is important to ensure that it is administered to patients with low-risk profiles and any side effects can be carefully mitigated.

Atherosclerosis is the underlying cause of many major CVDs, including stroke [10], peripheral artery disease [11], ischemic cardiomyopathy [12] and myocardial infarction [10]. The initial step of atherosclerotic plaque formation is endothelial cell (EC) dysfunction and low density lipoprotein (LDL)-cholesterol accumulation in the intima layer of arteries [10, 13]. Once internalized into cells, LDL-cholesterol is hydrolyzed into cholesterol which can accumulate as lipid droplets [14]. Lipid droplet accumulation can also be driven by cholesterol biosynthesis. In this process, low cholesterol levels in the endoplasmic reticulum (ER) membrane activate the sterol regulatory element binding transcription factor 2 (SREBP2), which transcriptionally upregulates LDL receptor (LDLR) and HMG-CoA synthase [14]. Whether fluoxetine impacts these mechanisms in ECs to promote early atheroscleorotic disease development remains unclear.

In this study, we mechanistically dissect how fluoxetine impacts cholesterol metabolism in primary human ECs. We uncover fluoxetine-mediated dysregulation of cholesterol homeostasis, LDL uptake and lipid droplet accumulation through activation of the SREBP2 transcription factor.

## Materials and Methods

### Cell culture

HUVECs (C-12200 and C-12203, PromoCell, Germany) and HCAECs (C-12221, PromoCell, Germany) were cultured under hypoxia at 3% O_2_ from passages 3 to 10. HUVECs were cultured on plates coated with 0.5% gelatine (48723, Sigma-Aldrich, Germany) in endothelial cell growth medium (ECGM): Ham’s F12 (21765037, Gibco, MA, USA) supplemented with 10% FBS (S0615, Sigma-Aldrich, Germany), 25 µg/ml heparin (A3004, AppliChem PanReac, Germany), 1 µg/ml ascorbic acid (A4403, Sigma-Aldrich, Germany), 2 mM GlutaMax (35050038, Gibco, MA, USA), 100 U/ml Penicillin-Streptomycin (15140122, Gibco, MA, USA) and endothelial cell growth supplement (ECGS, 02-102, Millipore, MA, USA). HCAECs were cultured with MesoEndo cell growth medium (212-500, PromoCell, Germany). For confocal imaging, ECs were cultured in Millicell EZ Slides (PEZGS0896, Sigma-Aldrich, Germany) pre-coated with 10 µg/ml fibronectin (33010018, Gibco, USA). Treatments were performed in full media with 20 µM fluoxetine [15] (sc-201125, Santa Cruz, TX, USA), 1 µM simvastatin (S6196, Sigma-Aldrich, Germany), 20 µM sertraline (S6319, Sigma-Aldrich, Germany), 20 µM citalopram (C7861, Sigma-Aldrich, Germany), 1 µM U18666A (HY-107433, MedChemExpress, USA), 1 µM hydroxycholesterol (sc-214091, Santa Cruz, TX, USA) or DMSO (A3672, AppliChem PanReac, Germany) prior to analyses.

### Viability assay

Media from fluoxetine treated cells was saved into a collection tube. Cells were washed twice with PBS, and the discarded PBS was transferred to the same collection tube. Cells were trypsinised and harvested into the same collection tubes, and centrifuged (300 xg, 5 min). The pellet was resuspended in 500 µl 2% FCS and stained with 0.5 µl 0.1 mg/ml propidium iodide (PI) (P2864, Sigma-Aldrich, Germany). PI fluorescence intensity was measured with the BD Fortessa instrument. Data was analysed with FlowJo^TM^ v10.8.2. Cells with a positive PI signal were classified as dead.

### Bulk RNA sequencing and analysis

HUVECs were treated for 12 h with 20 µM fluoxetine or DMSO (vehicle). Cells were washed twice with cold PBS and the RNA was extracted with the miRNeasy Mini Kit (217004, Qiagen, Netherlands) according to the manufacturer’s protocol. The RNA integrity number (RIN) was measured using the LabChip GX Touch Nucleic Acid Analyzer (Revvity, MA, USA) and the concentration was measured using Qubit. RNA samples with a RIN value > 7 were selected for mRNA sequencing (poly-A selected). The libraries were prepared using the Illumina TruSeq stranded mRNA library preparation kit following the manufacturer’s instructions. After a final QC, the libraries were sequenced in paired-end mode (2×100 bases) in the Novaseq6000 sequencer (Illumina, CA, USA) in an S2.200 flow cell with a depth of more than 30 million reads per sample.

### RNA seq data analysis

Raw sequencing reads were initially processed for quality control and pre-processing using fastp (v0.23.3) [16], which involved trimming adapter sequences, removing low-quality bases, and eliminating poly-G tail contamination. The quality of the reads was subsequently evaluated with FastQC (v0.11.4) [17] to confirm data integrity. The cleaned reads were then aligned to the human reference genome (GRCh38.p13) [18] using the STAR aligner (v2.5.2b) [19]. Alignment was performed with default parameters, permitting up to 50 multiple alignments per read to account for multi-mapping. Gene-level quantification was conducted with featureCounts (v2.0.1) [20], utilizing options suitable for reverse-stranded libraries. A fractional counting approach was employed to accurately assign reads that mapped to multiple locations, ensuring precise quantification of gene expression levels. Differential gene expression analysis was performed with the R package DESeq2 (v1.42.1) [21] and data were normalized using the variance stabilization transformation (VST).

Upregulated genes were assigned as >0.5 log_2_ fold change (log_2_FC) and downregulated genes as log_2_FC < -0.5, with an adjusted *p*-value <0.05 cut-off. Pathway enrichment analyses were performed with the EnrichR package v(3.2). The databases used were BioPlanet [22] and TRRUST [23]. Additional graphs were made with the *ggplot2* v(3.5.1) package.

### Neutral lipid assessment

For confocal imaging, cells were cultured in Millicell EZ Slides and treated with 20 µM fluoxetine overnight. The slides were washed with PBS, fixed with 300 µl ROTI^®^Histofix (2213.4, Carl Roth, Germany) for 15 min and washed 3 times. The cells were then stained with 200 µl 10 µg/ml BODIPY™ 493/503 (D3922, Thermo Fisher Scientific, MA, USA) for 15 min and washed with PBS. Excess PBS was removed, and the slides were mounted on Mowiol (0713.1, Carl Roth, Germany) with DAPI (A3672, AppliChem PanReac, Germany). Images were obtained with a Zeiss LSM 980 with Airyscan 2 confocal microscope and analysed with ImageJ.

For flow cytometry measurement, cells were harvested via trypsinisation, washed with PBS and the supernatant was discarded. The cells were then resuspended in the remaining supernatant and fixed with 250 µL ROTI^®^Histofix for 30 min on ice. Cells were washed twice with PBS, resuspended in the remaining supernatant and stained with 200 µl 10 µg/ml BODIPY™ 493/503 for 15 min at room temperature. Cells were washed twice with PBS and resuspended in 250 µl 2% FCS. BODIPY intensity was measured with the BD LSR Fortessa instrument. Data was analysed with FlowJo^TM^ v10.8.2.

### Lipidomics

Lipidomics were carried out by MetaSysX GmbH, Germany. HUVECs were treated with fluoxetine for 24 h and washed 3 times with ice-cold PBS. Metabolites were extracted in three rounds. First, 500 µl of ice-cold 80% methanol was added to the cells, they were scraped thoroughly, and the extract was transferred into a pre-chilled tube. The same procedure was repeated with 100% methanol and then 100% isopropanol. All extracts were transferred to the same collection tube. Samples were centrifuged, the cell pellet discarded, and the supernatant dried in a vacuum concentrator.

Sample preparation was performed as previously described [24]. The samples were measured with a Waters ACQUITY Reversed Phase Ultra Performance Liquid Chromatography (RP-UPLC) coupled to a Thermo-Fisher Exactive mass spectrometer. For the lipophilic measurements C8 columns were used. Chromatograms were recorded in Full Scan MS mode (Mass Range [100-1500]). All mass spectra were acquired in positive and negative ionization modes. Extraction of the LC-MS data was accomplished with the software PeakShaper (metaSysX GmbH, Germany). Alignment and filtration of the LC-MS data were completed using in-house software. The alignment of the extracted data from each chromatogram was performed according to the criteria that a feature had to be present in all replicates of at least one of the groups. The alignment of the data was followed by the application of various filters to refine the dataset, which included the removal of (i) isotopic peaks, (ii) in-source fragments of the analytes (due to the ionization method), and (iii) redundant peaks. The annotation of the content of the sample was performed by database query of mass-to-charge ratio and the retention time of detected features within certain criteria. An in-house database of chemical compounds was used for annotation.

For GC-MS, the samples were measured on an Agilent Technologies GC coupled to a Leco Pegasus HT mass spectrometer. Column: 30 meters DB35; Starting temp: 85 °C for 2 min; Gradient: 15 °C per min up to 360 °C. Samples were measured in splitless mode, injection of full volume. NetCDF files that were exported from the Leco Pegasus software were imported to “R”. The Bioconductor package TargetSearch [25] was used to transform retention time to retention index (RI), to align the chromatograms, to extract the peaks, and to annotate them by comparing the spectra and the RI to the Fiehn Library and to a user created library. The annotation of peaks was manually confirmed in Leco Pegasus. Analytes were quantified using a unique mass. Metabolites with an RT and a mass spectra that did not result in a match in the database were kept as not assigned metabolites.

Normalization was performed for each platform separately. The normalized intensities were merged as a final data matrix. Data was normalized to the median of intensities of the whole experiment (Sample Median Normalization, SMN).

### NanoSIMS – sample preparation

HUVECs were cultured and treated with fluoxetine for 24 h, washed with PBS and detached by adding 0.02% trypsin-EDTA (TE) for 1 min. The trypsin was stopped by 10% FBS (S0615, Sigma-Aldrich, Germany) containing media, and the detached cells were centrifuged at 300 xg for 5 min and resuspended in 100 µl of media. 4 µl of resuspended cells were pipetted into a gold-plated copper planchet type A (H-shaped) and topped with the flat side of a type B planchette (U-shape) (HPF ø3mm carrier type-A 16770141/type-B 16770142, Leica, Germany). The stack of planchettes was placed in a carrier cartridge (3 mm half cylinders, 16770135; middle plate, 16770136, Leica, Germany) and then high pressure frozen in a Leica HPM100 machine. The type A planchette was then placed face up into a sample holder (reagent bath 16707154; flow-through rings 16707157, Leica, Germany) of the Leica AFS2 freeze substitution machine. The cells were first fixed by 0.2% glutaraldehyde in freeze substitution media (90% acetone 10% bidest. H_2_O) for 72 h at -90 °C followed by a temperature increase to -45 °C, washed 3 times for 30 min with pure acetone and infiltrated with K4M (Lowicryl^TM^ K4M polar Kit, freshly prepared) resin undergoing another temperature increase to -25 °C. The resin was polymerised via UV whilst being subjected to the third temperature increase to 0 °C. After polymerization the resin block was trimmed and 500 nm sections were cut with the ultramicrotome (EM UC7, Leica, Germany). The sections were sputter coated with a 10 nm Au/Pd (80/20%) layer (EM SCD500, Leica, Germany) to reduce sample charging and to make the surface conductive. This sample was analysed via nanoSIMS (CAMECA nanoSIMS 50 L, AMETEK, France).

### NanoSIMS – analysis

The analysis of intracellular fluorine distribution was implemented with a NanoSIMS 50 L instrument (AMETEK, CAMECA, France). Secondary ion species (^19^F^−^, ^12^C_2_^–^, ^12^C^13^C^–^, ^12^C^14^N^−^, ^13^C^14^N^−^, ^31^P^−^ and ^32^S^−^) were detected with mass-resolving power (MRP = M/dM) of ~9000 achieved with 20×140 μm (width x height) nominal size of the entrance slit, 40×1800 µm exit slits, 200 × 200 μm aperture and an energy slit cutting off 20% of secondary ions in their energy-distribution tail. Pre-implantation with 16 keV caesium was performed with 200 pA ion Cs^+^ beam over 60 × 60 µm^2^ area for 10 min prior to analysis. Fields of view (FoV) of 20 × 20 µm^2^ up to 40 × 40 µm^2^ within the pre-implanted area were analysed in a 512×512 pixel raster using a 3 pA primary Cs^+^ beam with a dwelling time of 2 ms/pixel. Data acquired in 50 scans were processed with the open-source Look@NanoSIMS software [26]. The data acquired with each scan were corrected for the lateral drift recognized in the secondary electron intensity map (Esi) and all detected planes were accumulated for each ion species. The ^12^C^14^N^−^ maps were used for cell recognition, and ^32^S^−^ and ^31^P^−^ maps - for the identification of intracellular compartments.

### Limited proteolysis

Human umbilical vein endothelial cells were used at passage 1. Proteins were extracted using 1× PBS through homogenization and separated into 6 parts. Three parts received solvent and three parts were treated with fluoxetine hydrochloride 500 µmol/L. Samples were processed as previously described [27]. Following tryptic digestion at 37 °C for 16 h, desalting and lyophilization, peptides were redissolved in 0.1% formic acid (FA). 2 μg of peptides from each fraction were spiked with internal standard peptides and analysed using data-independent acquisition (DIA) mass spectrometry (Mobile phases Solvent A: 0.1% formic acid in water and Solvent B: 0.1% formic acid in 84% acetonitrile) using a Q Exactive HFX hybrid quadrupole-Orbitrap mass spectrometer (Thermo Fisher Scientific, MA, USA). Data were directly imported into Spectronaut software (Spectronaut™ 14.4.200727.47784, Biognosys, Switzerland) to build the spectral library using the human_uniprot database.

Peptides with a *p*-value <0.05 were considered enriched. Pathway enrichment analysis was performed with the respective gene IDs of enriched peptides. The EnrichR package v(3.2) was used with the KEGG database. The volcano plot was made with the *ggplot2* v(3.5.1) package.

### Immunoblotting

Protein lysates were prepared in a RIPA buffer (150 mM NaCl, 1% NP-40, 0.5% Sodium deoxycholate, 0.1% SDS and 50 mM Tris pH 7.5) supplemented with PhosSTOP (04906837001, Roche, Switzerland) and cOmplete™ Protease Inhibitor Cocktail (11836145001, Roche, Switzerland). Samples were loaded into 4-12% gradient SDS-Page gel and transferred to a PVDF membrane (88520, Thermo Fisher Scientific, MA, USA). Membranes were blocked with 5% Skim milk or 2% BSA and incubated with antibodies overnight (Supplementary Table 4). Membranes were washed and incubated with a secondary HRP-conjugated antibody for 2 h (Supplementary Table 4). Membranes were developed with either Lumi-Light (12015200001, Roche, Switzerland) or SuperSignal™ West Femto (34095, Thermo Fisher Scientific, MA, USA) and detected on a GBox Syngene instrument. Protein quantification was carried out with ImageJ.

### LDL Uptake

Cells were treated with 20 µM fluoxetine overnight, washed twice with PBS and incubated with 20 µg/ml Dil-LDL (L3482, Thermo Fisher Scientific, MA, USA) diluted in 1% fatty acid free BSA (A8806, Sigma-Aldrich, Germany) in Ham’s F12. After 6 h incubation, cells were washed twice with PBS to remove excess Dil-LDL and harvested. Dil intensity was measured with the BD Fortessa instrument. Data were analysed with FlowJo^TM^ v10.8.2.

For confocal imaging, cells were cultured in EZ Millicell slides and treated with fluoxetine for 24 h. Cells were washed with PBS and incubated for 2 h with 0.2 mg/ml anti-LDLR antibody (AF2255, R&D Systems, MN, USA) or mouse IgG (12-371, Sigma-Aldrich, Germany) in ECGM. Following the incubation, cells were washed with PBS and treated with 20 µg/ml Dil-LDL in ECGM overnight. The next day, cells were washed twice and fixed with ROTI^®^Histofix for 15 min at room temperature. The slides were washed 3 times with PBS and mounted with Mowiol + DAPI. Images were obtained with a Zeiss LSM 980 with Airyscan 2 confocal microscope (Zeiss, Germany) and analysed with ImageJ.

### Immunostaining

HUVECs were cultured in 8 well Millicell EZ Slides (PEZGS0896, Millipore, Germany) and treated with fluoxetine 20 µM for 24 h. Cells were fixed with a 1:1 dilution of ROTI^®^Histofix (P087.1, Carl Roth, Germany) in complete cell culture medium (400 µl) for 5 min, washed with PBS, and fixed again with 200 µl ROTI^®^Histofix for 15 min. Cells were washed twice with PBS and blocked with either 10% normal goat serum (50197Z, Life technologies, CA, USA) or 5% donkey serum (S30-M, Sigma-Aldrich, Germany) for 1 h at room temperature. Cells were incubated overnight at 4 °C with either anti-SERCA2 antibody (1:100) (ab150435, Abcam, UK) or anti-LDLR antibody (1:200) (AF2255, R&D Systems, MN, USA) diluted in 1% normal goat serum and 1% donkey serum, respectively. After two washes with PBS, cells were incubated for 2 h with AlexaFluor 488 goat anti-rabbit IgG antibody (1:400) (A11034, Thermo Fisher Scientific, MA, USA) or AlexaFluor 555 donkey anti-goat IgG antibody (1:400) (A32816, Thermo Fisher Scientific, MA, USA) in PBS. After washing in PBS, slides were mounted on Mowiol (0713.1, Roth) containing DAPI (A3672, AppliChem PanReac). Images were obtained with a confocal microscope (LSM980, Zeiss, Germany) and analysed with ImageJ (v1.54p).

### Puromycin incorporation experiments

HUVECs were cultured in 6-well plates until confluency and treated with fluoxetine 20 µM for 24 h. Cells were washed with PBS and incubated with 10 µg/ml Puromycin (A1113803, Life Technologies, MA, USA) for 10 min followed by two washes with PBS. Cells were lysed with RIPA buffer (150 mM NaCl, 1% NP-40, 0.5% Sodium deoxycholate, 0.1% SDS and 50 mM Tris pH 7.5) supplemented with PhosSTOP (04906837001, Roche, Switzerland) and cOmplete™ Protease Inhibitor Cocktail (11836145001, Roche, Switzerland). Puromycin incorporation was evaluated by immunoblotting.

### Cell cycle

HUVECs (50,000 cells) were cultured in 12-well plates and treated with vehicle or 20 µM fluoxetine for 24 h. Cells were then trypsinized, centrifuged and the supernatant was removed. The cell pellet was gently resuspended in the residual supernatant. Cells were fixed and permeabilized with the BD Cytofix/Cytoperm™ Fixation/Permeabilization Kit (554714, BD Biosciences, NJ, USA). First, 250 µl of Fixation/Permeabilization solution was added and cells were incubated for 30 min on ice. Cells were washed twice with 1 ml 1× BD Perm/Wash™ buffer and centrifuged. The pellet was resuspended in 200 µl DAPI (10 µg/ml) and incubated for 30 min at room temperature. Cells were then washed twice with 2 ml of 2% FCS in PBS. DAPI fluorescence intensity was measured using a BD LSRFortessa instrument. Data were analysed with FlowJo^TM^ 10.8.2. G1 and G2 peaks were identified in the DAPI intensity histogram, and S-phase cells were quantified as the population between the peaks.

### Endothelial migration

HUVECs (50,000 cells) were seeded in uncoated 8 chamber slides (80806, Ibidi, Germany) and cultured to confluency. A longitudinal scratch was introduced using a 200 µl pipette tip. Chambers were washed with Dulbecco’s PBS and cells treated with 20 µM fluoxetine in full EGM-2 (C-22011, Promocell, Germany) media. Cells were imaged using an IncuCyte live-cell imaging system (Essen Bioscience, Germany) which automatically captured 16 images per chamber every 2 h for 48 h. Endothelial migration was analysed with ImageJ.

### Sprouting assay

The protocol was performed as previously described [28] with minor modifications.

A stock solution was prepared using 1.6% methyl cellulose (M0512, Sigma-Aldrich, Germany) in EGM-2 media (C-22011, Promocell, Germany). A total of 455,000 HUVECs were suspended in 13.6 ml medium, and 1.4 ml of the 1.6% methyl cellulose stock solution was added. After gentle mixing, the cell suspension was pipetted into multiple 25 µl drops on a 10-cm square Petri dish to generate hanging drops, which were incubated upside down to allow spheroid formation. Spheroids were collected with 6 ml of 10% FBS in PBS and centrifuged at 200 xg for 8 min at room temperature. Meanwhile, the collagen solution was prepared on ice containing 12.45 mM HEPES (H0887, Sigma-Aldrich, Germany), 32.4 mM NaHCO₃ (25080-060, Gibco, NY, USA), 4.2 µg/ml Rat Tail Collagen Type 1 (354236, Corning, NY, USA) and 4 mM NaOH. After centrifugation, spheroids were resuspended in 1.2 ml methocel stock solution. Rapidly, 1.2 ml of collagen solution was added to the spheroids and gently mixed. A total of 300 µl spheroid suspension was transferred to each well of a 48-well plate and incubated for 30 min at 37 °C to allow polymerization. EGM-2 media (100 µl) containing treatments (20 µM fluoxetine or DMSO) was added on top of the gels. Spheroids were incubated for 24 h in a hypoxia incubator (5% O_2_, 5% CO_2_, 37 °C). Gels were then washed with PBS and fixed with 100 µl ROTI^®^Histofix (P087.1, Carl Roth, Germany) for 1 h.

Spheroids were imaged by phase contrast microscopy. Individual sprouts were manually traced using ImageJ. Sprout lengths were corrected according to the microscope’s resolution.

### Oxidative stress assessment

Cells were treated with 20 µM fluoxetine or DMSO overnight, washed with PBS and incubated with 5 µM DCFH-DA (35845, Sigma-Aldrich, Germany) in Ham’s F12 for 30 min. Cells were washed 3 times with PBS and harvested for flow cytometry. DCFH-DA intensity was determined with the BD Fortessa instrument. Data was analysed with FlowJo^TM^ v10.8.2.

The MitoSOX™ Mitochondrial Superoxide assay was performed according to the manufacturer’s protocol. Briefly, cells were treated with 20 µM fluoxetine or vehicle overnight, washed with PBS and incubated with MitoSOX Red reagent (M36007, Thermo Fisher Scientific, MA, USA) for 30 min in PBS. Cells were washed 3 times with PBS and harvested for flow cytometry. MitoSOX Red intensity was determined with the BD Fortessa instrument. Data was analysed with FlowJo^TM^ v10.8.2.

### Free and esterified cholesterol quantification

Cells were treated with 20 µM fluoxetine overnight, washed with PBS and lysed in RIPA buffer (150 mM NaCl, 1% NP-40, 0.5% Sodium deoxycholate, 0.1% SDS and 50 mM Tris pH 7.5). Cholesterol quantifications were performed with the Amplex™ Red Cholesterol-Assay-Kit (A12216, Thermo Fisher Scientific, MA, USA) according to the manufacturer’s protocol. Total protein concentration of cell lysates was used to normalize cholesterol quantification values.

### Quantitative PCR

RNA was extracted with the miRNeasy Mini Kit (217004, Qiagen, Netherlands) and genomic DNA was digested with the RNase-Free DNase Set (79254, Qiagen, Netherlands) according to the manufacturer’s protocol. cDNA synthesis was performed with the Maxima First Strand cDNA Synthesis Kit (K1671, Thermo Scientific, MA, USA) per the manufacturer’s instructions. Power SYBR™ Green PCR Master Mix (4368708, Thermo Scientific, MA, USA) was used for the RT-qPCR. Oligonucleotide sequences are provided in Supplementary Table 5.

### Cholesterol efflux

HUVECs were seeded in 12-well plates and cultured until confluency. Cells were incubated for 18 h in endothelial cell growth medium containing 10% lipid-depleted FCS and 5 µM 22-NBD Cholesterol (SML3831, Sigma-Aldrich, Germany) in the presence or absence of fluoxetine 20 µM. Lipid-depleted FCS was prepared by incubating FCS with lipid removal adsorbent (LRA) (13360-U, Sigma-Aldrich, Germany). Briefly, 1 g LRA was added to 25 ml FCS and incubated overnight at 4 °C on a rotating wheel (40–60 rpm), followed by sequential centrifugation steps at 4000 rpm to remove the adsorbent. The supernatant was collected and sterile-filtered.

After labelling, cells were washed twice with PBS to remove excess 22-NBD cholesterol and incubated with fresh ECGM for 4 h. Medium from cells (100 µl) was transferred to a 96-well plate. Cells were then washed with PBS and lysed with 250 µl RIPA at 4 °C for 20 min on a rocking shaker. Cell lysates (100 µl) were transferred to a 96-well plate. Samples were diluted with 100 µl absolute ethanol and fluorescence was measured at 463 nm excitation and 536 nm emission. Background values were subtracted from all samples. Cholesterol efflux was calculated as the percentage of 22-NBD cholesterol in the medium relative to the total fluorescence detected in the corresponding cell lysates.

### siRNA gene knockdown

A 6% dilution of Lipofectamine™ RNAiMAX (13778150, Thermo Fisher Scientific, MA, USA) was prepared in Opti-MEM medium (51985026, Thermo Fisher Scientific, MA, USA). ON-TARGETplus siRNA pools targeting *SREBP2* (L-009549-00-0005, Horizon, CO, USA) and *SOAT1/ACAT1* (L-005240-00-0005, Horizon, CO, USA), as well as a non-targeting control pool (D-001810-10-05, Horizon, CO, USA) were diluted to 500 nM or 1000 nM in Opti-MEM media. The diluted lipofectamine and siRNA solutions were mixed at a 1:1 ratio and incubated for 5 min at room temperature to allow complex formation.

For *SREBP2* knockdown assays, siRNA complexes and fluoxetine were added simultaneously. Treatment medium containing 20 µM fluoxetine or DMSO and 10% siRNA complex solution was prepared in full ECGM medium, resulting in the final siRNA concentration of 25 nM. Cells were incubated with siRNA and fluoxetine for 24 h prior to analyses.

For *SOAT1/ACAT1* knockdown assays, cells were first incubated with 10% siRNA complex solution in OptiMEM for 6 h, resulting in the final siRNA concentration of 50 nM. The medium was then replaced with ECGM containing 20 µM fluoxetine, cells were incubated for an additional 18 h prior to analysis.

### LDL transcytosis assay

HUVECs were seeded in Transwell^®^ cell culture inserts with 0.4 µm pores (CLS3413, Corning, NY, USA) at 60,000 cells/well in a 300 µl suspension. Trans-endothelial electrical resistance (TEER) measurements were performed daily with the Millicel-ERS Volthometer (MERS00002, Millipore, Germany) and Milicell-ERS probe (MERSSTX01, Millipore, Germany) to evaluate barrier integrity. On day 2, cells were treated with 20 µM fluoxetine or vehicle and incubated overnight. The next day, tracers were added to the top chamber: 20 µM Dil-LDL and 16 µM 40 kDa FITC-Dextran (FD40S, Sigma-Aldrich, Germany). Samples of 100 µl were collected from the bottom well at 15, 60 and 240 min after the addition of tracer dyes. Dil-LDL (554/590) and FITC-Dextran (483/530) fluorescence intensity were measured in a VantaStar microplate reader.

### Statistical analysis

Data was analyzed using GraphPad Prism v10.0. Data are presented as mean ± sem. Data were analyzed using two-tailed Student’s *t*-tests, one-way ANOVA and two-way ANOVA, as indicated in the figure legends. For all experiments, at least 3 different biological replicates were used, and no blinding was undertaken. A biological replicate is defined as endothelial cells isolated from a different human donor(s). The precise number of biological replicates per experiment are indicated in the figure legends. No randomization was done to calculate sample size.

## Results

### Fluoxetine alters endothelial cell proliferation and angiogenesis

As EC dysfunction is a central feature of CVDs, we assessed the impact of fluoxetine on ECs. We cultured human umbilical vein endothelial cells (HUVECs) and treated them with 20 µM fluoxetine, which is comparable to its levels in the human brain following chronic fluoxetine intake [15]. This treatment did not affect cell viability (Supplementary Fig. 1A). RNA-seq of HUVECs revealed a significant downregulation of pathways related to cell cycle and mitosis (Supplementary Fig. 1B-C, Supplementary Table 1). Consistently, fluoxetine induced cell cycle arrest with ECs accumulating in the G1 phase (Supplementary Fig. 1D). In contrast, fluoxetine did not affect EC migration (Supplementary Fig. 1E), but significantly reduced sprout count in a spheroid angiogenesis assay indicating anti-angiogenic activity (Supplementary Fig. 1F). Together, these data indicate that fluoxetine impacts EC cell cycle and angiogenesis.

### Fluoxetine upregulates cholesterol metabolism in ECs

**Fig. 1:**
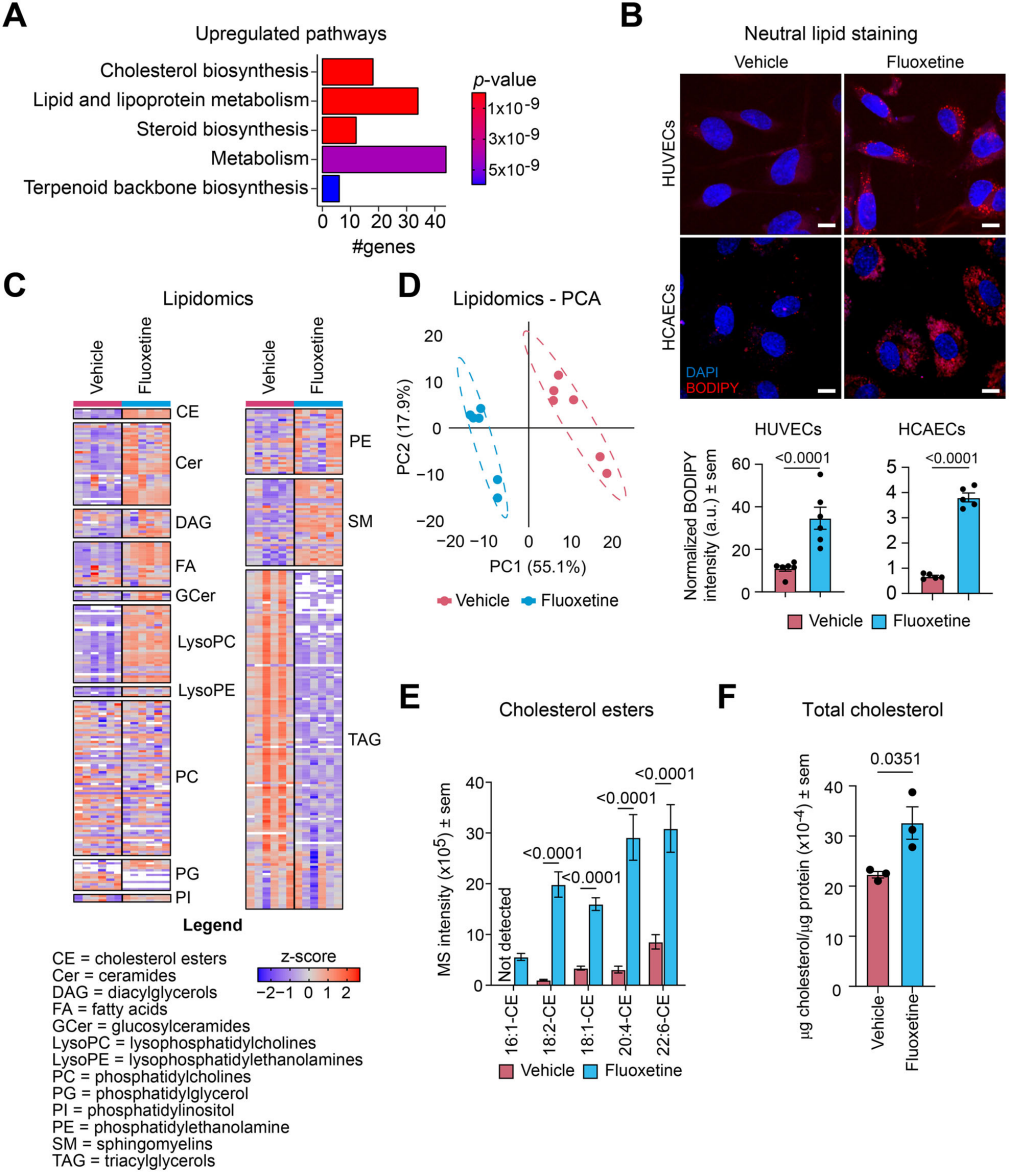
Fluoxetine disrupts cholesterol metabolism in ECs. **A** Bioplanet-annotated pathways enriched amongst upregulated genes in HUVECs treated with fluoxetine (20 µM). Fisher’s exact test *p*-value is provided. **B** Confocal microscopy images of BODIPY 493/503 staining of fluoxetine-treated ECs and their quantification. HUVECs: n = 6 biological replicates; HCAECs n = 5 biological replicates. Scale bars = 10 µm. **C** Heatmap of lipid species in HUVECs treated with fluoxetine or vehicle. Z-scores and legends are provided. **D** PCA analysis of lipidomics data. n = 6 biological replicates per group. **E** Relative abundance of cholesterol esters in fluoxetine- vs vehicle-treated HUVECs from the lipidomics data. n = 6 biological replicates per group. **F** Quantification of cholesterol (free and esterified) in HUVECs treated with fluoxetine or vehicle. n = 3 biological replicates per group. Cholesterol content (free and esterified) is normalized to protein concentration. Data are represented as mean values ± sem and were analysed with a two-sided Student’s *t*-test (**B**, **F**) and two-way ANOVA test (**E**). Biological replicates refer to ECs isolated from different human donor(s). CE, cholesterol esters; Cer, ceramides; DAG, diacylglycerols; FA, fatty acids; GCer, glucosylceramides; HCAECs, human coronary artery endothelial cells; HUVECs, human umbilical vein endothelial cells; LysoPC, lysophosphatidylcholines; LysoPE, lysophosphatidylethanolamines; MS, mass spectrometry; PC, phosphatidylcholines; PG, phosphatidylglycerol; PI, phosphatidylinositol; PE, phosphatidylethanolamine; SM, sphingomyelins (sphingolipids); TAG, triacylglycerols.

Amongst the upregulated genes in the RNA-seq analyses, cholesterol biosynthesis, and lipid and lipoprotein metabolism were the most significant upregulated pathways following fluoxetine treatment (Fig. 1A, Supplementary Fig. 1G). In contrast, inflammatory genes did not show strong changes (Supplementary Fig. 1H). To investigate the effects of fluoxetine on cholesterol and lipid metabolism, we undertook a range of metabolic assays. Consistent with our RNA-seq data (Fig. 1A), HUVECs and human coronary artery ECs (HCAECs) significantly increased lipid droplet accumulation following fluoxetine treatment (Fig. 1B). To systematically quantify changes in lipid species following fluoxetine administration, we performed lipidomic profiling using liquid chromatography-mass spectrometry (Supplementary Fig. 1I, Supplementary Table 2). We observed a global change in lipid profiles, with an increase in cholesterol esters, ceramides, fatty acids and sphingolipids, and a significant decrease in triacylglycerols in fluoxetine treated HUVECs (Fig. 1C-D). In comparison to other lipid species, cholesterol esters displayed the strongest increase, with some cholesterol esters showing an 8-fold increase following fluoxetine administration (Fig. 1E). We confirmed the increased levels of cholesterol and its esters in HUVECs via a fluorescence-based assay (Fig. 1F). Together, our data show that fluoxetine disrupts lipid metabolism, promotes lipid droplet formation, and increases accumulation of cholesterol esters in ECs.

### Fluoxetine promotes cholesterol biosynthesis and LDL uptake

**Fig. 2:**
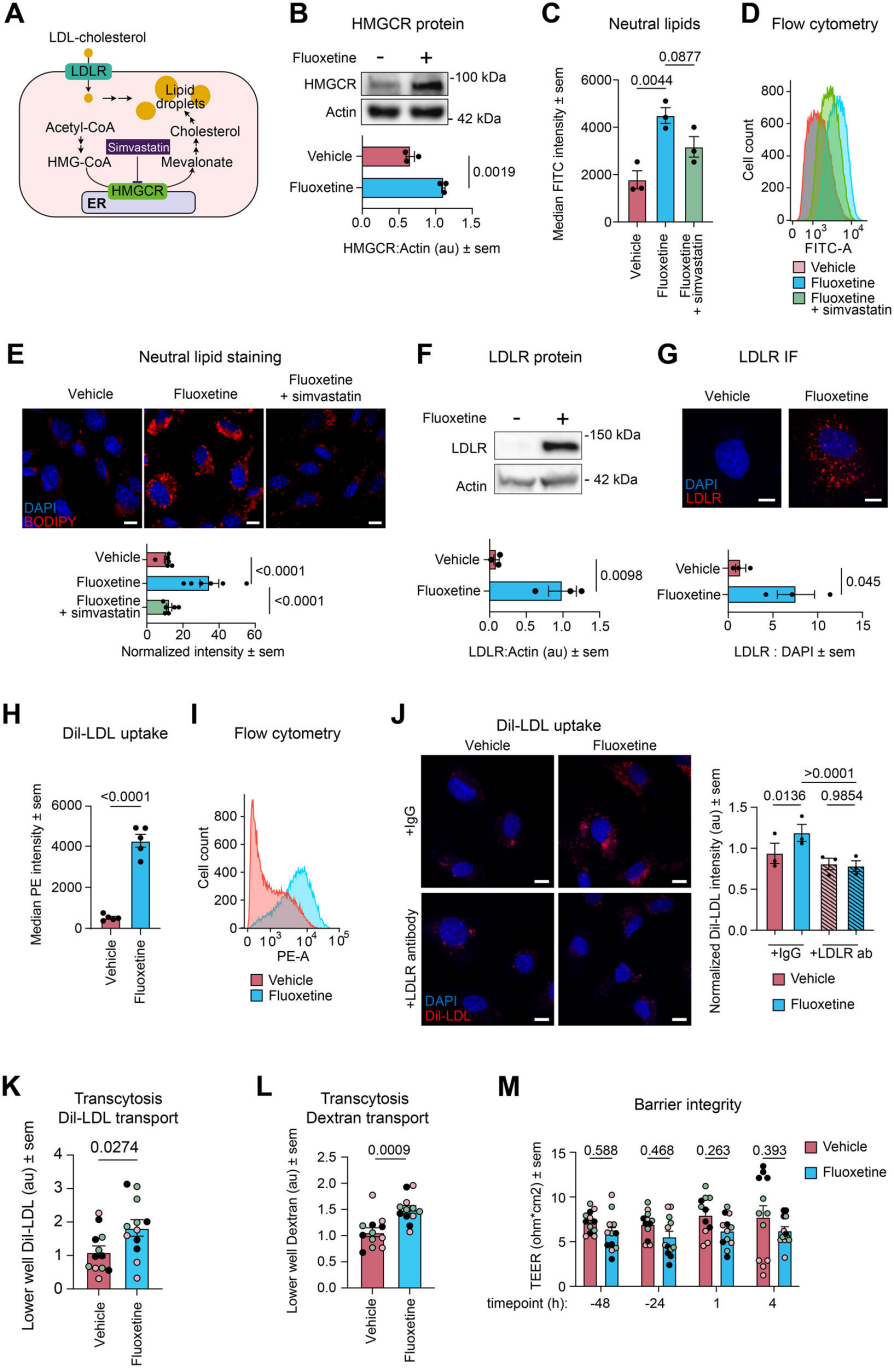
Fluoxetine promotes cholesterol biosynthesis and LDL uptake. **A** Diagram depicting cholesterol uptake via LDLR and the major cholesterol synthesis pathway. The statin simvastatin inhibits HMGCR, the rate limiting enzymatic step during cholesterol production. **B** Immunoblot of HMGCR in HUVECs treated with fluoxetine. β-actin is provided as a loading control. HMGCR intensities were normalized to β-actin. n = 3 biological replicates per group. **C–D** Flow cytometry analysis and quantification of BODIPY 493/503 in HUVECs treated with fluoxetine, simvastatin or vehicle. n = 3 biological replicates per group. **E** Neutral lipid quantification in HUVECs via BODIPY 493/503 and DAPI staining. Cells were treated with fluoxetine, simvastatin or vehicle. n = 6 biological replicates per group. Scale bars = 10 µm. **F** LDLR immunoblot of HCAECs treated with fluoxetine. β-actin is provided as a loading control. LDLR intensities were standardised to β-actin. n = 3 biological replicates per group. **G** Immunostaining of LDLR in HUVECs treated with fluoxetine for 24 h. Quantification of LDLR intensity is displayed below. n = 3 biological replicates. Scale bar = 10 µm. **H–I** Flow cytometry measurement and representative histograms of Dil-labelled LDL uptake by HUVECs after fluoxetine treatment. n = 5 biological replicates per group. **J** Confocal microscopy of Dil-labelled LDL uptake in HUVECs. Cells were pre-treated with fluoxetine or vehicle followed by treatment with IgG or anti-LDLR antibody. n = 3 biological replicates per group. Scale bar = 10 µm. **K** Fluorescence intensity of Dil-LDL transported from the top to the bottom chamber in a transcytosis model. Dot colours represent biological replicates per group (n = 3). **L** Measurement of 40 kDa FITC-Dextran transport from the top to the bottom chamber in a transcytosis model. Dot colours represent biological replicates per group (n = 3). **M** Transendothelial electrical resistance measurements across HUVECs monolayer in a transcytosis model before and after Dil-LDL and Dextran tracing. Dot colours represent biological replicates per group (n = 3). The data are presented as mean ± sem and were analysed with a one-way ANOVA test (**C**, **E**, **J**, **M**) or a two-sided Student’s *t*-test (**B**, **F**, **G**, **H**, **K**, **L**). Biological replicates refer to ECs isolated from different human donor(s). HMGCR, 3-hydroxy-3-methylglutaryl-coenzyme A reductase; IF, immunofluorescence staining; LDL, low density lipoprotein; LDLR, LDL receptor.

Building on our observation that fluoxetine increases lipid droplet and cholesterol accumulation in HUVECs (Fig. 1B-F), we next investigated whether this effect depends on cholesterol biosynthesis. HMGCR is the rate-limiting enzyme of cholesterol biosynthesis [14] (Fig. 2A), and HMGCR protein and *HMGCR* mRNA were significantly upregulated following fluoxetine treatment (Fig. 2B, Supplementary Fig. 1G). To test whether enhanced cholesterol biosynthesis contributes to lipid droplet accumulation, we treated HUVECs with either fluoxetine alone or in combination with simvastatin (SVT), a commonly used inhibitor of HMGCR [14] (Fig. 2A). As expected, fluoxetine led to increased lipid droplets and neutral lipids as identified via BODIPY staining and flow cytometry (Fig. 2C-E). Simvastatin partially attenuated lipid droplet formation in fluoxetine treated HUVECs (Fig. 2C-E), suggesting that an increase in cholesterol biosynthesis is partially responsible for enhanced lipid droplet formation in fluoxetine-treated cells.

Apart from increased synthesis, LDL receptor (LDLR)-mediated cholesterol uptake represents another mechanism leading to cholesterol accumulation [14]. *LDLR* mRNA and LDLR protein were significantly upregulated after fluoxetine treatment (Fig. 2F-G, Supplementary Fig. 1G). This was associated with higher uptake of fluorescently labelled LDL (Dil-LDL) in fluoxetine-treated HUVECs (Fig. 2H-I). To determine if this increase was mediated by LDLR, we targeted cell surface LDLR by treating ECs with an anti-LDLR antibody not permeable to the membrane. We found that the blocking of cell surface LDLR using anti-LDLR antibody treatment significantly attenuated fluoxetine-induced Dil-LDL uptake (Fig. 2J), suggesting that increased cholesterol accumulation following fluoxetine treatment is partially due to LDLR-mediated uptake of LDL.

We next considered whether the observed LDL uptake led to its transport across the vascular wall. As LDL transport across ECs is facilitated by transcytosis [29], we quantified Dil-LDL passage in a transcytosis model. We observed increased LDL transport across an endothelial monolayer (Fig. 2K), and higher cholesterol efflux from HUVECs following fluoxetine treatment (Supplementary Fig. 2A). Fluoxetine treatment also promoted increased transport of dextran molecules across the HUVEC monolayer (Fig. 2L), suggesting a general state of elevated transcytosis. However, fluoxetine did not significantly disrupt the endothelial barrier as shown by transendothelial electrical resistance measurements (Fig. 2M). Together, these data suggest that fluoxetine increases LDL transport across the EC layer via transcytosis without impacting the EC barrier.

### ACAT1 (SOAT1) is required for fluoxetine-mediated lipid droplet formation

We next considered mechanisms leading to lipid droplet formation in fluoxetine-treated ECs. Acyl coenzyme A:cholesterol acyltransferases (ACATs) catalyse the esterification of cholesterol [14], which allows cholesterol storage in intracellular lipid droplets. To determine whether fluoxetine-induced cholesterol ester accumulation depends on ACATs, we knocked down the *SOAT1* gene that encodes for the ACAT1 protein, the predominant ACAT isoform in HUVECs (Supplementary Fig. 2B). While fluoxetine led to increased levels of neutral lipids, *SOAT1* knockdown attenuated their accumulation (Supplementary Fig. 2C), indicating that ACAT1 is required for fluoxetine-mediated accumulation of cholesterol esters. In contrast, inhibition of the lysosomal cholesterol transporter Niemann–Pick type C protein 1 (NPC1) [14] with U18666A did not significantly affect neutral lipid levels in fluoxetine-treated cells (Supplementary Fig. 2D-F), suggesting that lysosomal cholesterol trafficking is not required for fluoxetine-induced cholesterol esterification.

Together, these data indicate that ACAT1 (SOAT1) is essential for the esterification of excess cholesterol and its incorporation into lipid droplets in fluoxetine-treated ECs.

### Fluoxetine localizes in the ER

**Fig. 3:**
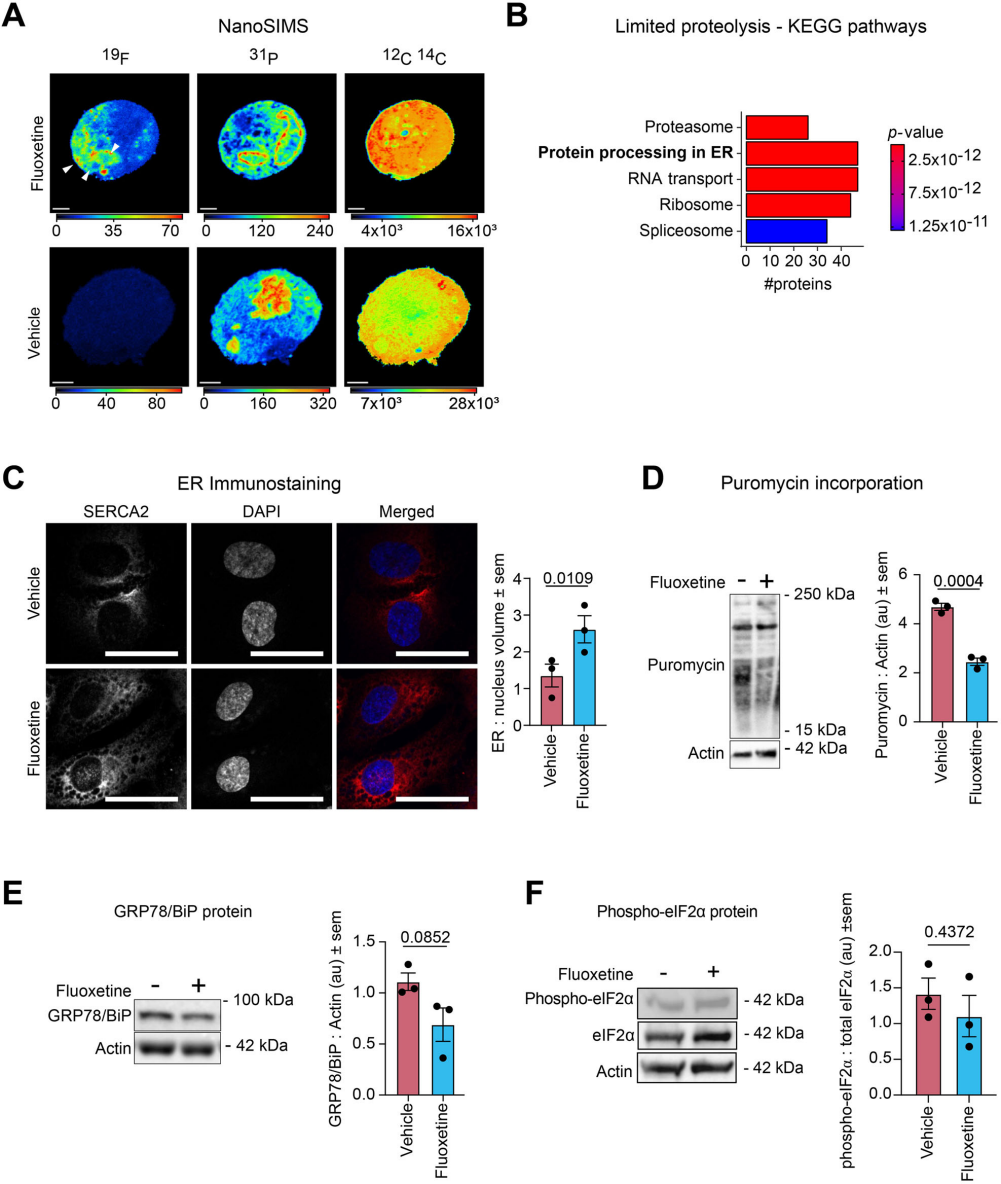
Fluoxetine localizes in the endoplasmic reticulum. **A** NanoSIMS images of ^19^F, ^31^P and ^12^C^14^N distribution in cells treated with vehicle and fluoxetine. Colours indicate ion intensities. Scale bars = 3 µm. Further images are provided in Supplementary Fig. 3. **B** KEGG-annotated pathways enriched amongst most abundant peptides in fluoxetine-treated cells versus controls. Fisher’s exact test *p*-values are provided. **C** Immunostaining of the ER marker *SERCA2* in HUVECs treated with fluoxetine. Quantification of ER volume is displayed on the right. n = 3 biological replicates. Scale bar = 10 µm. **D** Immunoblot of puromycin incorporation in nascent polypeptide chains of HUVECs treated with fluoxetine. β-actin is provided as a loading control. Anti-puromycin intensities were standardised to β-actin. n = 3 biological replicates per group. **E** GRP78/BiP immunoblot of HUVECs after fluoxetine treatment. β-actin is provided as a loading control. GRP78/BiP intensities were standardised to β-actin. n = 3 biological replicates per group. **F** Immunoblot analysis of eIF2α and phospho-eIF2α levels in HUVECs treated with vehicle or fluoxetine. eIF2α and phospho-eIF2α intensities were standardised to β-actin. n = 3 biological replicates per group. The data (**C**, **D**, **E**, **F**) are presented as mean ± sem and were analysed with a two-sided Student’s *t*-test. Biological replicates refer to ECs isolated from different human donor(s). BiP, binding immunoglobulin protein; ER, endoplasmic reticulum; GRP78, glucose-regulated protein 78.

To determine how fluoxetine triggers cholesterol biosynthesis and accumulation in ECs, we investigated where it accumulates in cells via nanoscale secondary ion mass spectrometry (NanoSIMS) [30, 31]. We measured fluorine (^19^F) ions to localize fluoxetine, as ^19^F is found in fluoxetine but its endogenous levels are very low in cells. Phosphorus (^31^P) is found in the DNA backbone and thus labels the nucleus. As expected, ^19^F was not detected in HUVECs in the absence of fluoxetine (Fig. 3A). In fluoxetine treated cells however, the ^19^F signal was substantially enriched around the nucleus (Fig. 3A, Supplementary Fig. 3A-B). These data suggest that fluoxetine localizes in the vicinity of the endoplasmic reticulum (ER) or the nuclear membrane.

To elucidate this further, we identified proteins interacting with fluoxetine in ECs via a limited proteolysis-mass spectrometry assay [27]. In this experiment, native protein extracts from HUVECs were incubated with fluoxetine, briefly digested, and analysed via mass spectrometry. We identified 2385 peptides with a significantly higher abundance in the fluoxetine-treatment group, corresponding to 1384 proteins (Supplementary Fig. 4A, Supplementary Table 3). As expected, known targets of fluoxetine in neurons, SERT [32] and TRKB [33], were not detected due to their low expression in ECs [34, 35]. KEGG pathway analysis of the enriched proteins indicated that fluoxetine primarily interacts with proteins in the ER (Fig. 3B). Together, the NanoSIMS and limited proteolysis assays suggest that fluoxetine localizes in the ER and interacts with proteins in this compartment.

### Fluoxetine leads to ER-expansion

The ER is a major hub for protein and lipid metabolism, and key enzymes required for lipid and cholesterol metabolism are located there [14, 36]. We evaluated whether fluoxetine affects ER morphology. Immunostaining revealed a significant expansion of the ER in fluoxetine-treated HUVECs (Fig. 3C), which is consistent with increased lipid synthesis. In contrast to the ER expansion, puromycin incorporation assays revealed reduced global translation rates in fluoxetine-treated HUVECs (Fig. 3D). Levels of the ER chaperone GRP78/BiP, and of phospho-eIF2α, did not show any significant changes following fluoxetine treatment (Fig. 3E-F). These data indicate that fluoxetine does not trigger ER stress in ECs. Furthermore, ROS and mitochondrial superoxide levels did not show any significant changes following fluoxetine treatment (Supplementary Fig. 4B-E).

Together, our data reveal that fluoxetine promotes ER expansion and lipid accumulation independently of oxidative and ER stress.

### Fluoxetine activates SREBP2 in ECs

**Fig. 4:**
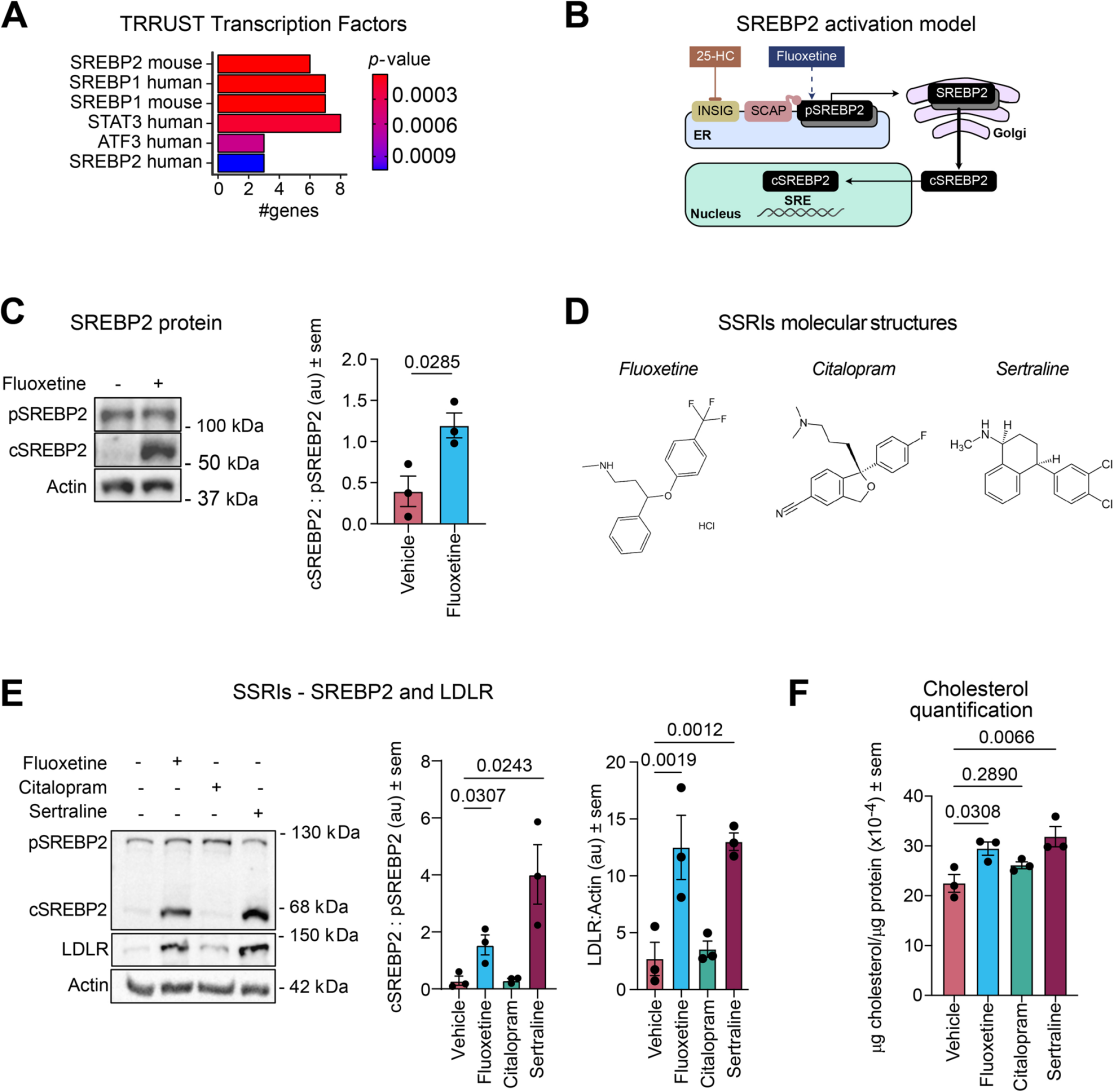
The SSRIs fluoxetine and sertraline activate SREBP2. **A** Enrichment of TRRUST-annotated transcription factors amongst top upregulated genes in fluoxetine-treated HUVECs. Fisher’s exact test *p*-values are provided. **B** Model depicting the SREBP2 activation mechanism in the ER and Golgi. **C** Cleaved (c) and precursor (p) SREBP2 immunoblot of HUVECs treated with fluoxetine. cSREBP2 and pSREBP2 intensities were standardised to β-actin. cSREBP2/pSREBP2 ratios are displayed on the right. n = 3 biological replicates per group. **D** Molecular structures of the SSRIs fluoxetine, citalopram and sertraline. **E** Immunoblot of cleaved (c) and precursor (p) SREBP2, LDLR and β-actin of HUVECs treated with fluoxetine (20 µM), citalopram (20 µM) or sertraline (20 µM). cSREBP2, pSREBP2 and LDLR intensities were standardised to β-actin. cSREBP2/pSREBP2 ratios are displayed on the right. n = 3 biological replicates per group. **F** Normalized quantification of free and esterified cholesterol in HUVECs treated with the SSRIs fluoxetine, citalopram or sertraline. n = 3 biological replicates per group. The data are presented as mean ± sem and were analysed with a two-sided Student’s *t*-test (**C**) or one-way ANOVA test (**E**, **F**). Biological replicates refer to ECs isolated from different human donor(s). LDLR, low density lipoprotein receptor; SSRIs, selective serotonin reuptake inhibitors.

For an unbiased insight into fluoxetine-mediated mechanisms deregulating cholesterol and lipid metabolism, we evaluated transcription networks affected by fluoxetine. Cross-referencing our RNA-seq data from fluoxetine-treated ECs (Fig. 1A) with the Transcriptional Regulatory Relationships Unraveled by Sentence-based Text mining (TRRUST) [23] database, revealed that genes upregulated by fluoxetine were associated with the SREBP1 and SREBP2 transcription factor networks (Fig. 4A). The transcription factor SREBP2 is found in the ER, translocates to the nucleus upon activation, and transcriptionally upregulates genes associated with cholesterol synthesis and LDL uptake [14] (Fig. 4B). Consistent with our transcriptional analyses (Fig. 4A), fluoxetine treatment of HUVECs led to increased levels of cleaved SREBP2, indicating SREBP2 activation (Fig. 4C). Together, these data indicate that fluoxetine leads to the activation of SREBP2.

### Sertraline but not citalopram triggers activation of SREBP2

Given that fluoxetine belongs to the SSRI family of drugs, we wondered whether other SSRIs could also trigger activation of SREBP2 and accumulation of lipids in ECs. We treated HUVECs with the SSRIs fluoxetine, sertraline and citalopram (Fig. 4D). Treatment with fluoxetine and sertraline, but not citalopram, led to the activation of SREBP2 and increased LDLR levels (Fig. 4E). Consistently, fluoxetine and sertraline, but not citalopram, induced accumulation of free and esterified cholesterol in ECs (Fig. 4F). These data suggest that some other members of the SSRI family are also able to activate SREBP2, but this is not a common feature of all SSRIs.

### SREBP2 inhibition reverses fluoxetine-induced disruption of cholesterol metabolism

**Fig. 5:**
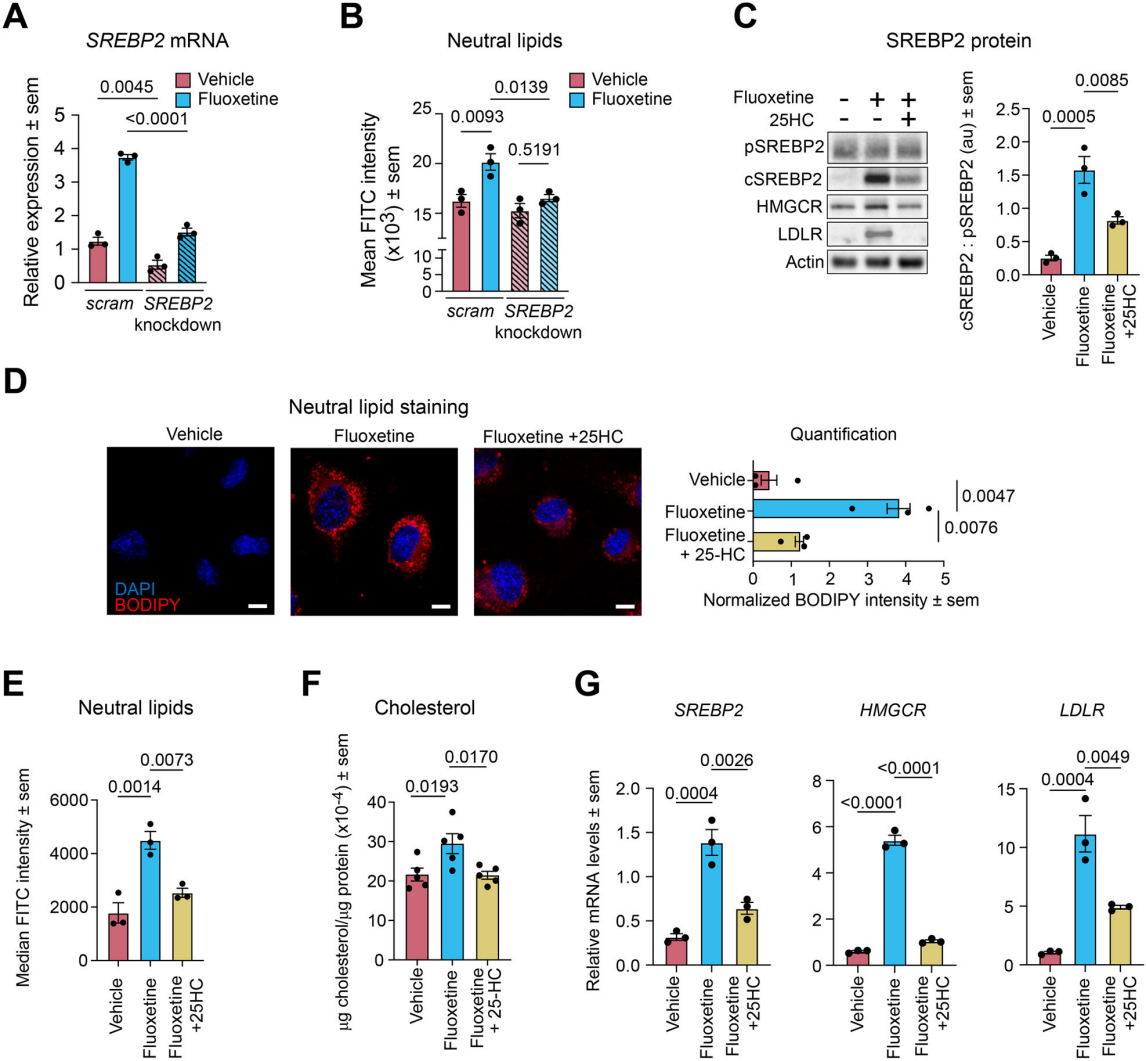
Fluoxetine promotes cholesterol accumulation via activation of SREBP2. **A** Relative *SREBP2* expression after a combination of *SREBP2* knockdown and fluoxetine treatment. n = 3 biological replicates per group. Gene expression was standardized to *GAPDH*. **B** Flow cytometry quantification of neutral lipids in HUVECs after *SREBP2* knockdown based on BODIPY 493/503 staining and measurement. n = 3 biological replicates per group. **C** Immunoblot of cleaved (c) and precursor (p) SREBP2, HMGCR, LDLR and β-actin in HUVECs treated with vehicle, fluoxetine and 25-HC. n = 3 biological replicates per group. cSREBP2 and pSREBP2 were first standardised to β-actin, and the cSREBP2 : pSREBP2 ratio subsequently calculated. HMGCR and LDLR standardized intensities are provided in Supplementary Fig. 5C-D. **D** Confocal microscopy images and quantification of neutral lipids in HUVECs after fluoxetine and 25-HC treatment via BODIPY 493/503 staining. n = 3 biological replicates per group. Scale bars = 10 µm. **E** Flow cytometry analysis of BODIPY 493/503 staining in HUVECs after fluoxetine and 25-HC treatment. n = 3 biological replicates per group. **F** Normalized quantification of cholesterol (free and esterified) in HUVECs treated with fluoxetine, 25-HC or vehicle. n = 5 biological replicates per group. **G** Relative gene expression of *SREBP2*, *HMGCR* and *LDLR* in HUVECs treated with a combination of fluoxetine and 25-HC. Gene expression levels were standardized to *GAPDH*. n = 3 biological replicates per group. The data are presented as mean ± sem and were analysed with one-way ANOVA test (**A**, **B**, **C**, **D**, **E**, **F**, **G**). Biological replicates refer to ECs isolated from different human donor(s). 25-HC, 25-hydroxycholesterol. HMGCR, 3-hydroxy-3-methylglutaryl-coenzyme A reductase; LDLR, low density lipoprotein receptor.

To understand if fluoxetine-mediated SREBP2 activation was relevant to the effects on cholesterol that we had observed, we knocked down *SREBP2* in HUVECs and treated them with fluoxetine (Fig. 5A). *SREBP2* knockdown significantly attenuated lipid droplet accumulation and reduced mRNA levels of the cholesterol metabolism enzymes *HMGCR, ACAT2* and *HMGCS1* in fluoxetine-treated HUVECs (Fig. 5B, Supplementary Fig. 5A-B). We additionally inhibited SREBP2 activation with 25-hydroxycholesterol (25-HC), a potent agonist of INSIG proteins [37] that in turn inhibits SREBP2 (Fig. 4B). 25-HC treatment prevented SREBP2 activation and reduced the levels of HMGCR and LDLR proteins in HUVECs treated with fluoxetine (Fig. 5C, Supplementary Fig. 5C-D). Consistently, 25-HC mitigated the activation of SREBP2 target genes *ACAT2, HMGCS1* and *FASN* in HUVECs and HCAECs, and significantly attenuated neutral lipid and cholesterol accumulation (Fig. 5D-G, Supplementary Fig. 5E). Together, these data uncover that SREBP2 activation by fluoxetine is necessary for the disruption of cholesterol metabolism in ECs.

## Discussion

Fluoxetine is a commonly prescribed antidepressant, administered to over 40 million patients. In this study, we uncovered that primary human ECs upregulate cholesterol biosynthesis and LDL-cholesterol uptake following fluoxetine treatment. Mechanistically, we find that fluoxetine localizes to the ER and activates the SREBP2 transcription factor, leading to increased lipid droplet formation in ECs. Overall, our data demonstrate that fluoxetine disrupts lipid metabolism in ECs and drives pathological changes that could potentially elevate the risk of cardiovascular diseases.

Animal studies have shown that fluoxetine accelerates the early development of atherosclerosis [9], however the mechanistic reasons are not clear. We found that fluoxetine leads to increased lipid droplet accumulation in ECs. Lipid droplet formation in the endothelium has recently been linked to faster atherosclerosis development [38]. Endothelial-specific ablation of *Atgl*, the rate-limiting enzyme for lipid droplet hydrolysis, results in the accumulation of triglyceride-rich lipid droplets in ECs of large arteries and an increased atherosclerotic plaque size in mice [38]. These findings demonstrate how disruptions in EC lipid metabolism can contribute to vascular disease. Our data suggest that fluoxetine treatment alone can disrupt lipid metabolism within ECs, promoting lipid droplet formation and thus potentially increasing the risk of atherosclerosis. Notably, fluoxetine triggered EC lipid accumulation in the absence of a lipid challenge, suggesting that it may induce EC dysfunction in the absence of traditional CVD risk factors.

Fluoxetine treatment upregulated LDLR in ECs and promoted LDLR-mediated lipoprotein uptake and transcytosis. The endothelium plays a direct role in the transport of lipids and lipoproteins to the tissues, mostly via transcytosis. The proteins facilitating LDL transcytosis in ECs include Caveolin-1, SR-BI and ALK1 [39, 40]. In contrast, LDLR only seems to play a role in EC-specific LDL uptake under certain conditions. For example, during inflammation, ECs treated with IL-1β display increased LDL transcytosis mediated by LDLR and Rab27a [41], and TNFα treatment similarly increases LDL transcytosis in ECs and upregulates LDLR [42]. However, the process we uncovered was not associated with inflammation, ER stress or oxidative stress, suggesting that fluoxetine-induced activation of SREBP2 is sufficient to stimulate LDL transport via LDLR, thereby revealing a new SREBP2-dependent mechanism for LDLR activation in ECs.

SREBP2 is a well-established regulator of cholesterol metabolism in mammalian cells; however, less is known about its function in ECs. Studies have demonstrated that EC activation of SREBP2 is closely related to a stressful EC environment, for example during oscillatory shear flow [43] and iron overload [44]. Inflammatory signalling via NF-κB also activates SREBP2 in ECs and consequently alters cholesterol metabolism [45]. ChIP-seq data confirmed that SREBP2 binds to the canonical cholesterol metabolism genes in ECs, including *HMGCR*, *LDLR* and *HMGCS1* [46]. Our data indicate that the SREBP2-dependent disruption of cholesterol metabolism by fluoxetine in ECs is similar to its effects in hepatocytes and glial cells [47, 48]. Similarly to ECs, fluoxetine promotes hepatic lipid accumulation in mice [47, 49] and in hepatocytes in vitro [49, 50]. Moreover, chronic fluoxetine intake can alter the lipid composition of juvenile Macaque brains, in particular polyunsaturated fatty acids [51]. In zebrafish, fluoxetine treatment promotes the deregulation of cholesterol metabolism genes in the brain [52]. These findings suggest that the effects of fluoxetine on lipid homeostasis are not exclusive to ECs but may extend across multiple tissues, potentially promoting systemic alterations in lipid metabolism.

In line with these systemic effects observed across experimental models, clinical studies have investigated whether fluoxetine and other SSRIs impact circulating lipid profiles in patients. Despite these studies, the effects of fluoxetine and other SSRIs on patients’ serum lipid profiles remain controversial. Several studies report that SSRI treatment in individuals with major depressive disorder [53], schizophrenia [54], bipolar disorder [54], panic disorder [55, 56], and other psychiatric conditions [57] is associated with increased levels of total cholesterol and LDL cholesterol. In particular, fluoxetine has been linked to elevated triglycerides, total cholesterol, and low-density lipoprotein levels [53]. In contrast, other studies suggest that fluoxetine and SSRIs exert neutral or even beneficial effects on lipid profiles [58, 59]. For instance, in overweight or obese patients, fluoxetine treatment has been associated with reductions in triglycerides, LDL, and total cholesterol [59–61], likely as a consequence of weight loss [62]. Taken together, these findings indicate that fluoxetine and SSRIs can have substantial but variable effects on serum lipids, highlighting the importance of periodic monitoring of lipid levels in patients treated with SSRIs.

In conclusion, we show here that fluoxetine activates SREBP2 and disrupts cholesterol and lipid metabolism in endothelial cells. These findings reveal a putative mechanism by which fluoxetine can contribute to the development of atherosclerotic disease.

## Supplementary information

Supplementary Figures

Supplementary Tables

## Data Availability

All sequencing data have been uploaded to NCBI GEO (GSE304246).
