# Supplementary material for: Fluoxetine disrupts cholesterol metabolism in endothelial cells via SREBP2 activation
Source: Transl Psychiatry. 2026 Jun 23;16:318. doi: 10.1038/s41398-026-04197-x (PMC13291327; doi:10.1038/s41398-026-04197-x)
Supplement: Supplementary file 1 — Supplementary Figures [file 41398_2026_4197_MOESM1_ESM.docx]

**Supplementary Figures to:**

# **Fluoxetine disrupts cholesterol metabolism in**

# **endothelial cells via SREBP2 activation**

Fabiana Oliveira, Christina Papa, Tobias Hagemann, Ruby Schipper,​ Florian Geier, Tino Röxe, Faiqa Zulfqar, Christoph Prönnecke, Lisa Schmidt, Hryhoriy Stryhanyuk, Anne Hoffmann, Anastasia Kyselova, Christina Karantanou, Yuli Buckley, Muhammad Asad Farhan, Jesús Rafael Rodríguez-Aguilera, Saira Ambreen, He Yao, Amna Arif, Hugo N. G. Martin, Thomas Ebert, Nora Klöting, Matthias Blüher, Khurrum Shahzad, Jes-Niels Boeckel, Carolina E. Hagberg, Rima Chakaroun, Sofia-Iris Bibli, Bilal N. Sheikh


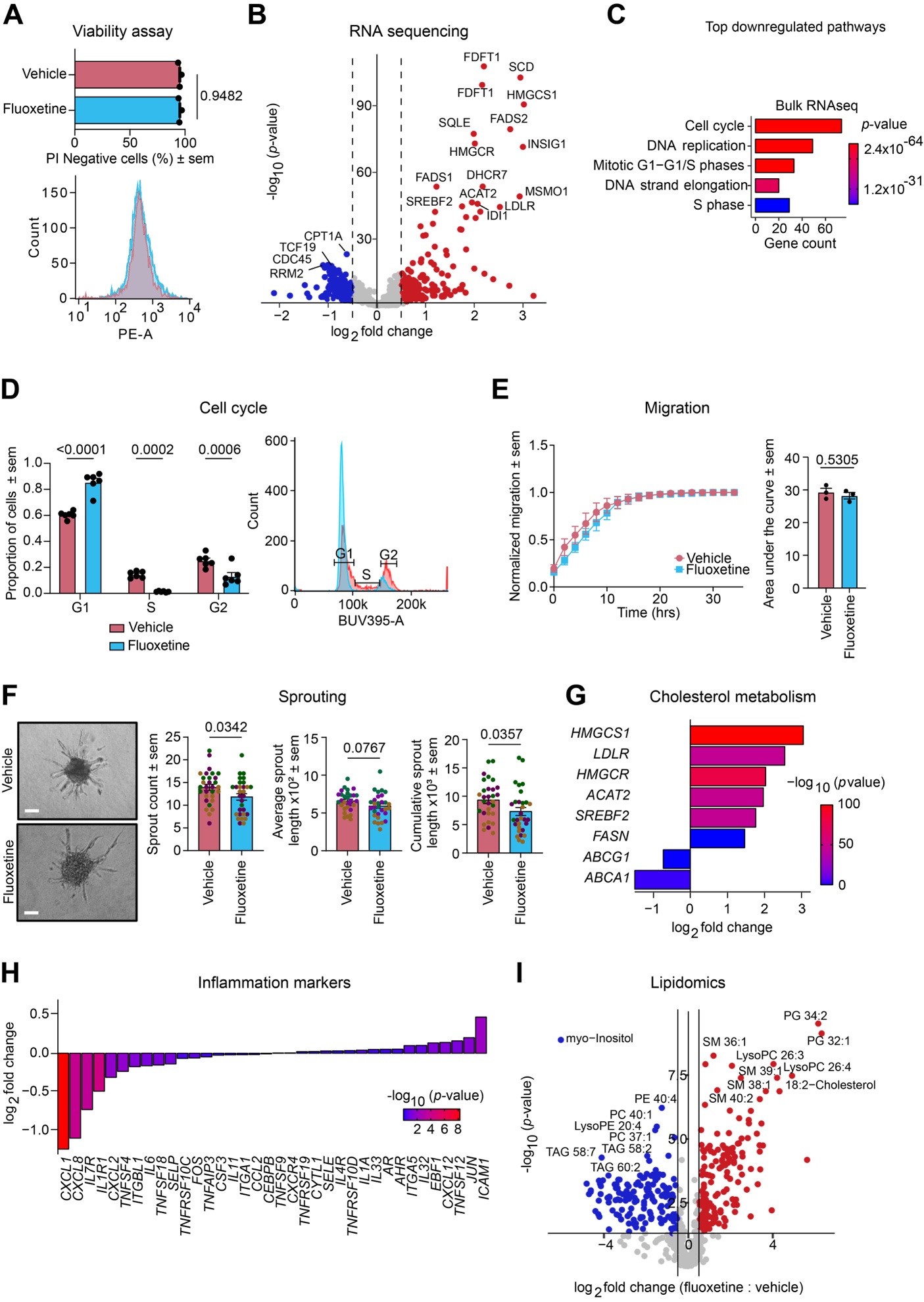


**Supplementary Figure 1 –** *Fluoxetine deregulates genes associated with cholesterol metabolism and cell cycle.* **A.** Flow cytometry quantification for viability of fluoxetine-treated HUVECs. Viable cells were defined as negative for propidium iodide staining. n *=* 3 biological replicates per group. **B.** Volcano plot of differentially expressed genes in HUVECs treated with fluoxetine. n *=* 4 biological replicates per group. Blue and red dots represent downregulated and upregulated genes, respectively. **C.** Bioplanet-annotated pathways of significantly downregulated genes in HUVECs following fluoxetine treatment. Fisher's exact test *p*-values are provided. **D.** Cell cycle distribution of HUVECs following 24-hour treatment with fluoxetine. Cell cycle phases were determined by quantifying DAPI intensity using flow cytometry. n *=* 6 biological replicates per group. Representative flow cytometry histogram demonstrating DAPI intensity is provided. **E.** Scratch wound assay of HUVECs treated with fluoxetine or vehicle. Migration of cells was followed by time‐lapse microscopy. Area under the curve values are shown on the right. n *=* 3 biological replicates per group. **F.** Endothelial cell sprouting in a modified spheroid assay. Spheroids were treated with vehicle or fluoxetine. Scale bar indicates 100 µm. Sprout count, average sprout length and cumulative sprout length are shown on the right. Dots correspond to individual spheroids. Colours represent biological replicates (n=3). **G.** Changes in expression of selected cholesterol metabolism genes in HUVECs following fluoxetine treatment. Data were derived from RNA-seq analyses. **H.** Changes in expression of selected inflammation marker genes in HUVECs following fluoxetine treatment. Data were derived from RNA-seq analyses. **I.** Volcano plot of relative lipid abundance in HUVECs after fluoxetine treatment. n *=* 6 biological replicates per group.

The data are presented as mean ± sem and were analysed with a two-sided Student’s *t*-test (**A, E, F**) or one-way ANOVA test (**D**). Biological replicates refer to ECs isolated from different human donor(s).


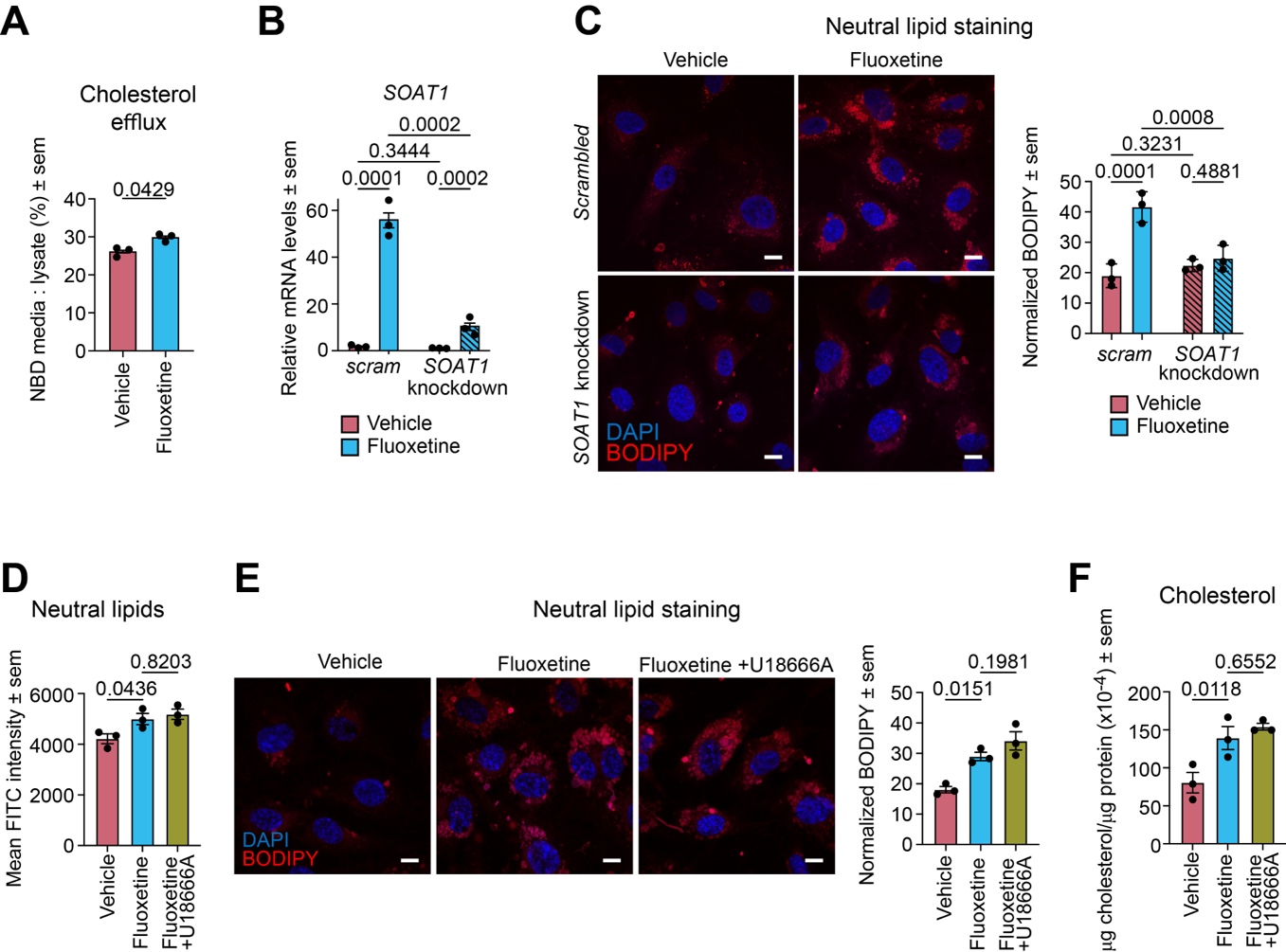


**Supplementary Figure 2 –** *ACAT1 is essential for the fluoxetine-mediated neutral lipid accumulation.* **A.** Cholesterol efflux measurements in HUVECs treated with fluoxetine. Cholesterol efflux was calculated using the fluorescence intensity of 22-NBD cholesterol in the medium relative to the cell pellet (%). n = 3 biological replicates per group. **B.** Relative gene expression of *SOAT1* (encodes for ACAT1 protein) in HUVECs after a combination of *SOAT1* knockdown and fluoxetine treatment. Gene expression levels were normalized to *GAPDH*. n = 3 biological replicates per group. **C.** Confocal microscopy of neutral lipids in HUVECs after a combination of *SOAT1* knockdown and fluoxetine treatment. Neutral lipids were stained with BODIPY 493/503. n = 3 biological replicates per group. Scale bars = 10 µm. **D.** Flow cytometry quantification of neutral lipids in HUVECs treated with a combination of fluoxetine and U18666A. Cells were stained with BODIPY 493/503. n = 3 biological replicates per group. **E.** Confocal microscopy of neutral lipids in HUVECs after a combination of fluoxetine and U18666A treatment. Cells were stained with BODIPY 493/503. n = 3 biological replicates per group. Scale bars = 10 µm. **F.** Normalized quantification of total (free and esterified) cholesterol in HUVECs treated with fluoxetine and U18666A. n = 3 biological replicates per group.

The data are presented as mean ± sem and were analysed with a two-sided Student’s *t*-test (**A**) or one-way ANOVA test (**B, C, D, E, F**). Biological replicates refer to ECs isolated from different human donor(s).


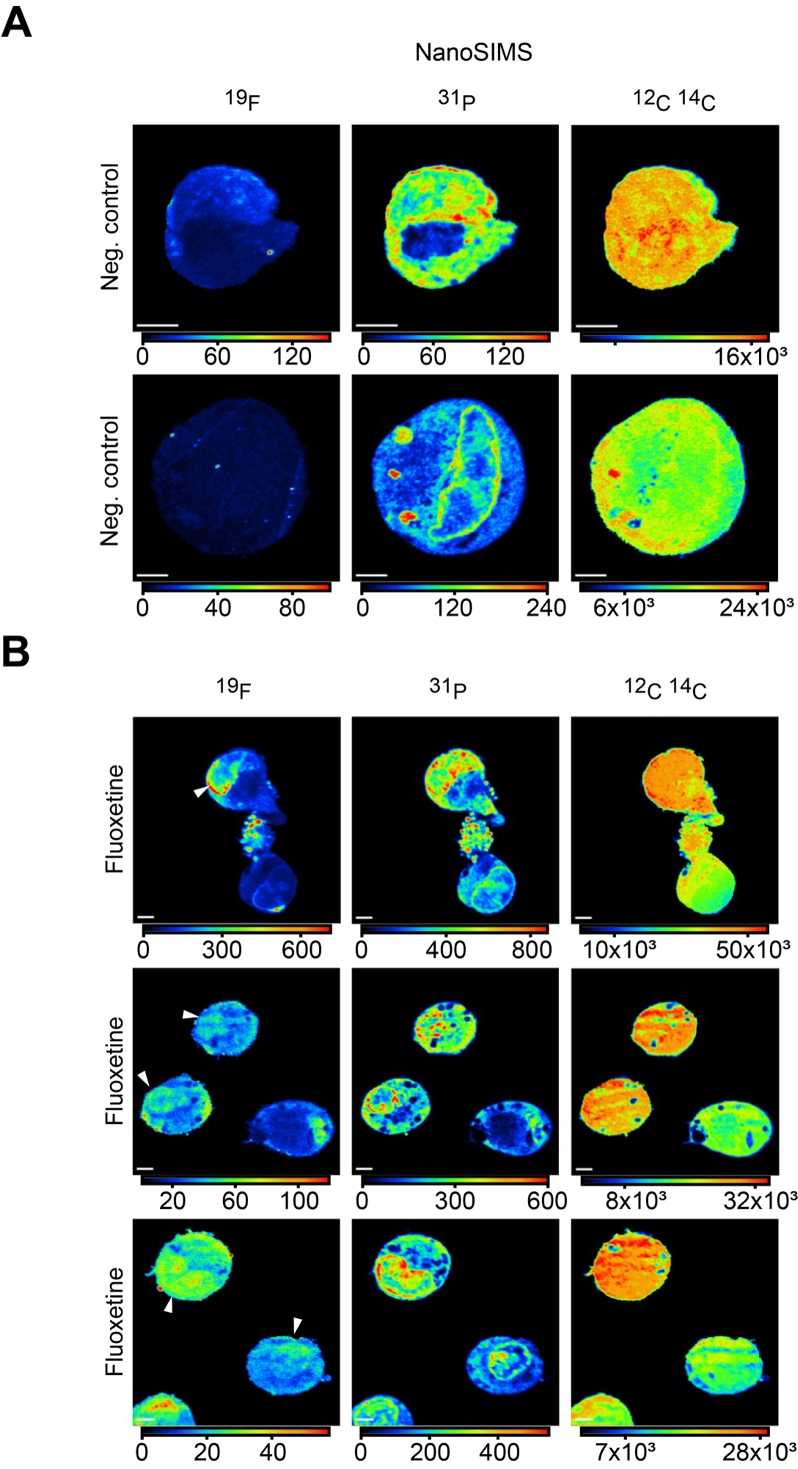


**Supplementary Figure 3 –** *Fluoxetine localizes in the peri-nuclear region.* NanoSIMS images of ^19^F, ^31^P and ^12^C^14^N distribution in cells treated with vehicle (**A**) and fluoxetine (**B**). Colours indicate ion intensities. Scale bars = 3 µm.


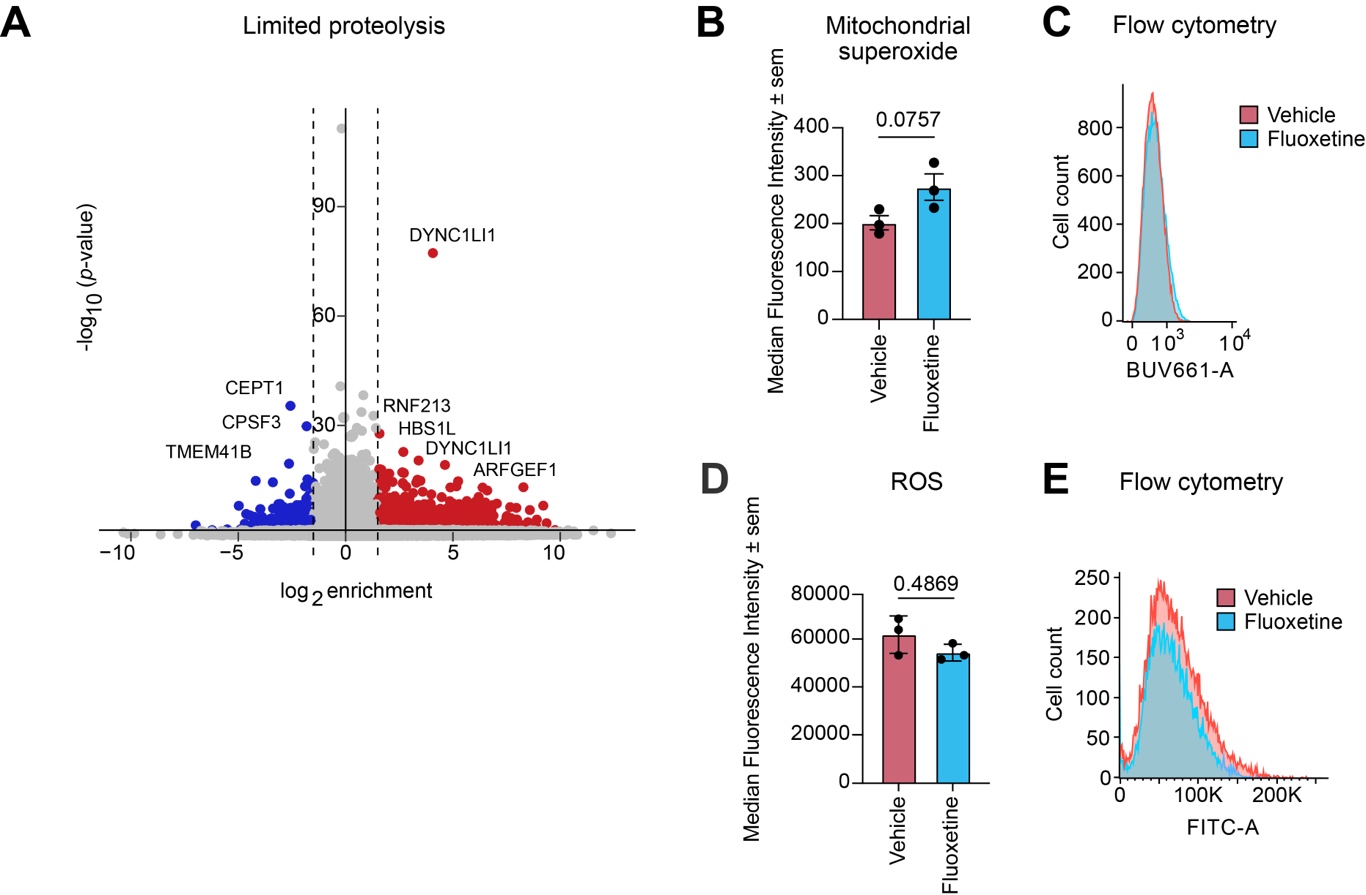


**Supplementary Figure 4** **–** *Fluoxetine does not impact ROS levels.* **A.** Volcano plot of limited proteolysis – mass spectrometry data. Log_2_ enrichment refers to peptide abundance in fluoxetine versus vehicle. **B-C.** Flow cytometry measurement of MitoSOX Red, a mitochondrial superoxide indicator, for fluoxetine treated HUVECs versus vehicle controls. n = 3 biological replicates per group. **D-E.** Flow cytometry analysis of the oxidative stress detector DCFH-DA in fluoxetine treated HUVECs. n = 3 biological replicates per group.

Data are presented as mean ± sem and were analysed with a two-sided Student’s *t*-test (**B, D**). Biological replicates refer to ECs isolated from different human donor(s). ER, endoplasmic reticulum; ROS, reactive oxygen species.


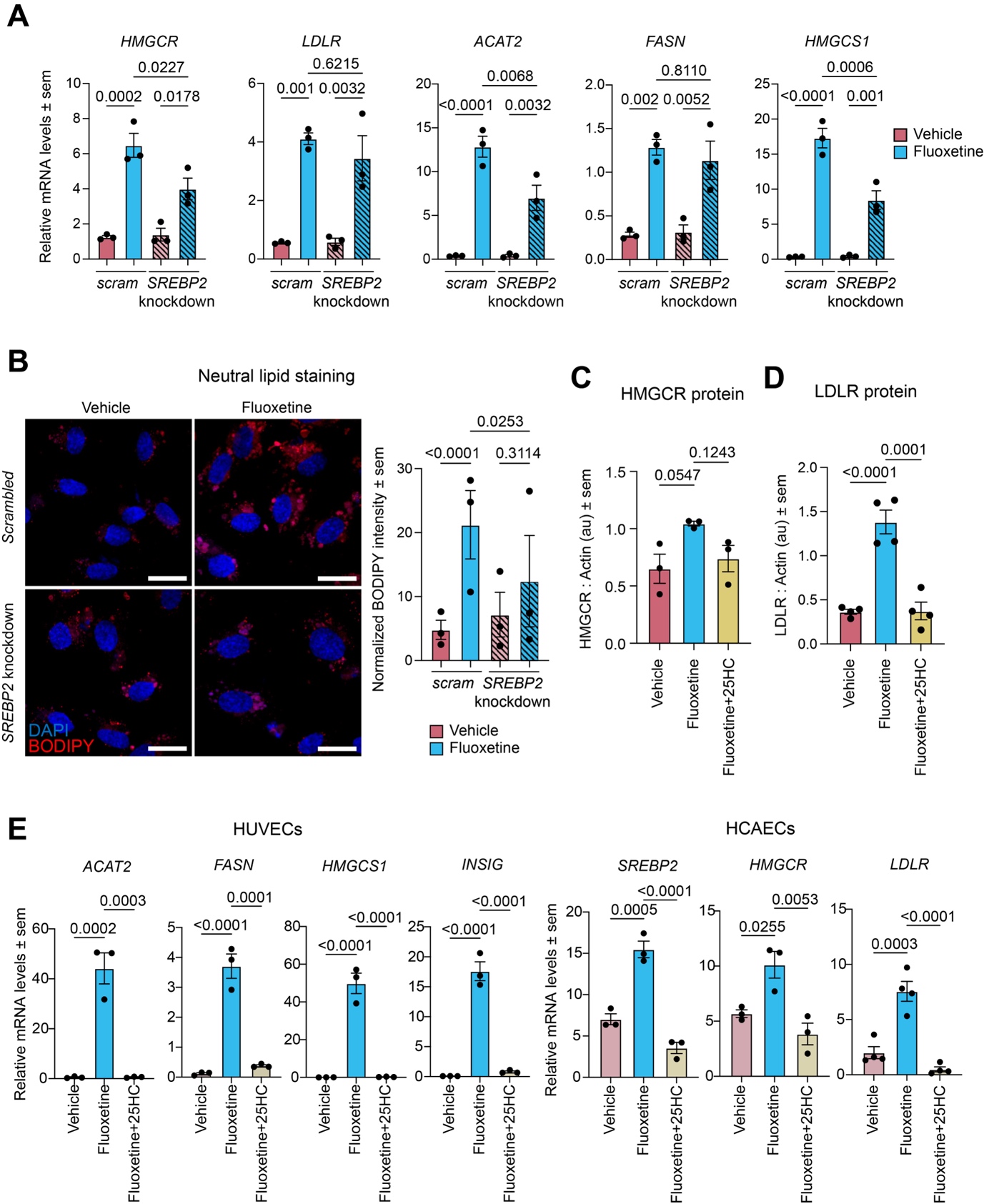


**Supplementary Figure 5 –** *SREBP2 inhibition attenuates the effects of fluoxetine.* **A.** Relative gene expression of *HMGCR*, *LDLR*, *ACAT2*, *FASN* and *HMGCS1* in HUVECs after a combination of *SREBP2* knockdown and fluoxetine treatment. Gene expression levels were normalized to *GAPDH*. n = 3 biological replicates per group. **B.** Confocal microscopy of neutral lipids in HUVECs after a combination of *SREBP2* knockdown and fluoxetine treatment. Cells were stained with BODIPY 493/503. n = 3 biological replicates per group. Scale bars = 10 µm. **C-D.** HMGCR (**C**) and LDLR (**D**) immunoblotting quantification of HUVECs treated with fluoxetine and 25-HC. The Western blot images are provided in Fig. 5C. n = 3 biological replicates per group. HMGCR and LDLR levels were normalized to β-actin. **E.** mRNA levels of select SREBP2-target genes in HUVECs and HCAECs treated with a combination of fluoxetine and 25-HC. Gene expression levels were normalized to *GAPDH*. n = 3 biological replicates per group.

Data are presented as mean ± sem and were analysed with a one-way ANOVA test (**A, B, C, D, E**). Biological replicates refer to ECs isolated from different human donor(s). 25-HC, 25-hydroxycholesterol; LDLR, low density lipoprotein receptor.
